# Oropharyngeal microbiome evaluation highlights *Neisseria* abundance in active celiac patients

**DOI:** 10.1038/s41598-018-29443-1

**Published:** 2018-07-23

**Authors:** Laura Iaffaldano, Ilaria Granata, Chiara Pagliuca, Maria Valeria Esposito, Giorgio Casaburi, Giuliana Salerno, Roberta Colicchio, Marina Piccirillo, Carolina Ciacci, Giovanna Del Vecchio Blanco, Mario Rosario Guarracino, Paola Salvatore, Francesco Salvatore, Valeria D’Argenio, Lucia Sacchetti

**Affiliations:** 1 Ceinge Biotecnologie Avanzate scarl, Naples, Italy; 2 LabGTP (Laboratory of Genomics, Transcriptomics and Proteomics), Institute for High Performance Computing and Networking (ICAR), National Research Council (CNR), Naples, Italy; 3 Department of Molecular Medicine and Medical Biotechnologies, University of Naples Federico II, Naples, Italy; 4 Task Force on Microbiome Studies, University of Naples Federico II, Naples and Ceinge Biotecnologie Avanzate scarl, Naples, Italy; 5 Department of Microbiology and Cell Science, University of Florida, Space Life Science Lab, Merritt Island, FL USA; 6 Department of Environmental, Biological and Pharmaceutical Sciences and Technologies, University of Campania Luigi Vanvitelli, Caserta, Italy; 7 Department of Medicine and Surgery, University of Salerno, Salerno, Italy; 8 Department of System Medicine, University of Rome Tor Vergata, Rome, Italy; 9 Present Address: Evolve Biosystems, Inc, Davis, CA 95618 USA

## Abstract

We previously profiled duodenal microbiome in active (a-), gluten-free diet (GFD) celiac disease (CD) patients and controls finding higher levels of the Proteobacterium *Neisseria flavescens* in a-CD patients than in the other two groups. Here, we investigate the oropharyngeal microbiome in CD patients and controls to evaluate whether this niche share microbial composition with the duodenum. We characterized by 16S rRNA gene sequencing the oropharyngeal microbiome in 14 a-CD, 22 GFD patients and 20 controls. Bacteroidetes, Proteobacteria and Firmicutes differed significantly between the three groups. In particular, Proteobacteria abounded in a-CD and *Neisseria* species mostly accounted for this abundance (p < 0.001), whereas Bacteroidetes were more present in control and GFD microbiomes. Culture-based oropharyngeal microbiota analysis confirmed the greater abundance of Proteobacteria and of *Neisseria* species in a-CD. Microbial functions prediction indicated a greater metabolic potential for degradation of aminoacids, lipids and ketone bodies in a-CD microbiome than in control and GFD microbiomes, in which polysaccharide metabolism predominated. Our results suggest a continuum of a-CD microbial composition from mouth to duodenum. We may speculate that microbiome characterization in the oropharynx, which is a less invasive sampling than the duodenum, could contribute to investigate the role of dysbiosis in CD pathogenesis.

## Introduction

Celiac disease (CD) is a complex autoimmune enteropathy triggered by ingestion of gluten in genetically susceptible individuals^1^, although alterations in the gut microbiome (virus and some bacterial strains) have also been implicated in its pathogenesis^2–5^. We recently reported, higher levels of Proteobacteria and lower levels of the Firmicutes and Actinobacteria phyla in the duodenal mucosal microbiome of patients suffering from active CD (a-CD) compared to CD patients on a gluten-free diet (GFD) and control subjects^3^. In detail, members of the *Neisseria* genus (*Neisseria flavescens* species, β-Proteobacteria class) were significantly more abundant in the microbiomes of a-CD patients than in the other 2 study groups, and provoked an inflammatory response in dendritic cells and in duodenal mucosal explants^3^. Our results, as well as other studies, indicate that gut dysbiosis exerts a role in mounting the immune response observed in a-CD patients^3,6–9^.

Microbiome alterations are best investigated in the duodenum because the alterations in intestinal architecture (mucosal atrophy and crypt hyperplasia), that are diagnostic for CD, occur in that tract^2^. However, duodenal samples are difficult to collect for ethical reasons and/or for scarce compliance of patients. Consequently, it would be useful to find an alternative that is equally informative but more easily obtained in order to investigate the role of the microbiome in CD. The gastrointestinal tract may be considered a single ecosystem extending from the oral cavity to the rectum, although eating habits and the conditions of the different niches influence the composition of the microbiome in the different sites. Notably, the CD trigger (i.e., gluten) is first processed in the mouth, and here the oral microbiota might have an impact on the immunogenic peptides produced after this first part of digestion process^10^. In this scenario, the objectives of our study were: (1) to characterize by 16S rRNA sequencing and by a culture-based approach the oropharyngeal microbiota in three groups: a-CD, GFD patients and control subjects; (2) to evaluate if the CD-associated oropharyngeal microbiome share the duodenal microbial composition as previously found^3^ and here profiled in a new subgroup of a-CD patients, which would suggest a continuum between these two niches^3^; and (3) to evaluate, by microbial functions prediction, if the metabolic potential is different in a-CD, GFD patients and controls. The identification of CD-associated microbial composition in oropharynx could contribute to deepen the role of the microbiome in the CD.

## Results

The general characteristics of the three groups enrolled in the study are listed in Table S1. Breastfeeding and vaginal delivery were referred by most subjects (>70%), and did not differ among the three groups. Similarly, mean age did not differ among the groups, whereas female gender was lower (p = 0.001) in a-CD than in GFD patients (Table S1).

### 16S rRNA sequencing of oropharyngeal and duodenal microbiomes

**Figure 1:**
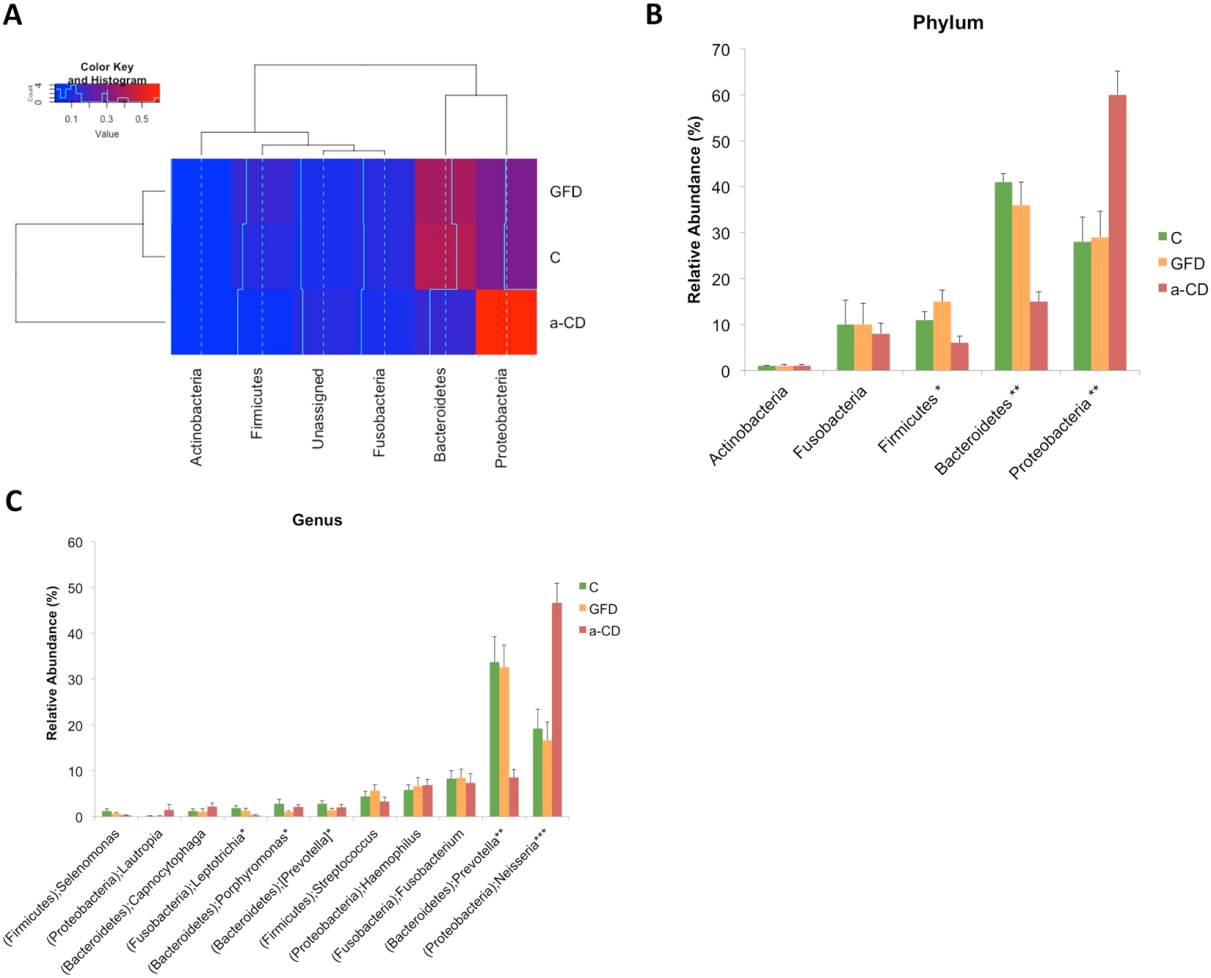
Hierarchical clustering and composition analysis of oropharyngeal microbiomes in the Control (C), gluten-free diet (GFD) and active celiac disease (a-CD) groups. (**A**) Heat map generated by using gplots R package of taxa relative abundances at phylum level. Phyla were retained if the relative abundance mean was ≥1% in at least one of the three groups under study. Rows and columns represented study groups and phyla, respectively. Both samples and phyla have been subjected to unsupervised hierarchical clustering and the results are depicted by the two dendrograms on the left and top of the image. Similarities between the control and GFD groups determined their grouping in one cluster, distinct from a-CD samples; the separation was mainly due to differences in Proteobacteria and Bacteroidetes phyla. Colors and histograms represent the abundances. (**B**,**C**) The barplots show the relative abundance (%) of taxonomic groups at phylum and genus level, according to the Greengenes database v.13_8. Phyla and genera having abundance greater than 1% in at least one group of study were reported. Error bars indicate standard error. Proteobacteria and Bacteroidetes were the most abundant phyla in all 3 groups. *Neisseria* was significantly more abundant (p < 0.001) in a-CD patients (46.6%) than in controls (19.2%) and GFD patients (16.6%). Phylum (p) to which the genus belongs is also reported. Statistical significance among the three groups was assessed by Kruskal Wallis test. Asterisks refer to the significance of differences among the three groups (*p < 0.05; **p < 0.005; ***p < 0.001).

Sample metadata obtained by 16S rRNA sequencing of the oropharyngeal microbiomes in the three study groups and in the duodenum of 7/14 a-CD patients are reported in Table S2. The weight of gender, age, and GFD period on the microbiome composition were investigated by Pearson correlation and no significant relationship were highlighted. As shown in Figs 1A and S1, the oropharyngeal microbiome differed between the C and GFD groups and the a-CD patients. In fact, GFD and control subjects clustered together in terms of phyla, distinguishing from a-CD samples; this separation was mainly due to differences in Proteobacteria and Bacteroidetes phyla as shown by hierarchical clustering dendrogram on phyla.

Five major bacterial phyla (OTU abundance ≥1%) populated the oropharynx of our study groups: Actinobacteria, Bacteroidetes, Firmicutes, Fusobacteria and Proteobacteria **(**Fig. 1B). The abundance of Bacteroidetes, Firmicutes and Proteobacteria differed significantly among the three groups (P < 0.05) (Fig. 1B). In detail, Bacteroidetes and Proteobacteria were the most abundant phyla in all study groups but, the Bacteroidetes/Proteobacteria ratio showed an opposite trend in a-CD samples with respect to C and GFD samples, namely a higher abundance of Proteobacteria at the cost of Bacteroidetes (Fig. 1B). Going from phylum to genus level in the two most abundant Bacteroidetes and Proteobacteria phyla (Fig. 1B,C**)**, the *Prevotella* (Bacteroidetes) and the *Neisseria* (Proteobacteria) genera were significantly less (p < 0.005) and more (p < 0.001) abundant in a-CD patients (8.52% and 46.63%, respectively) than in the other two groups (GFD: 32.63% and 16.67%, respectively; control: 33.75% and 19.23%, respectively) (Fig. 1C). Members of the Lachnospiraceae family (p-Firmicutes and Clostridiales order) were significantly less abundant (p < 0.001) in a-CD than in the other two groups (Fig. S2). In order to identify the exact pairwise comparisons, which gave rise to statistically significant different abundances, we performed the post-hoc Dunn’s test. It was confirmed that the significant differences were mainly due to the comparisons between a-CD and control groups, both healthy and GFD (Table S3).

The microbial profiles in the duodenum of a-CD patients were similar to those previously described in a different cohort of 20 a-CD patients^3^. In fact, the duodenal microbiome consisted of five major phyla (Bacteroidetes, Proteobacteria, Fusobacteria, Firmicutes and Actinobacteria), of which Proteobacteria was the most abundant (Fig. S3). The figure also shows a very similar microbiome composition in the oropharyngeal and duodenal samples from the same patients, apart from a higher abundance of Actinobacteria in the oropharynx than in the duodenum. Notably, Proteobacteria was the most abundant phylum in both niches (45% and 52% in duodenum and oropharynx, respectively) (Fig. S3).

**Figure 2:**
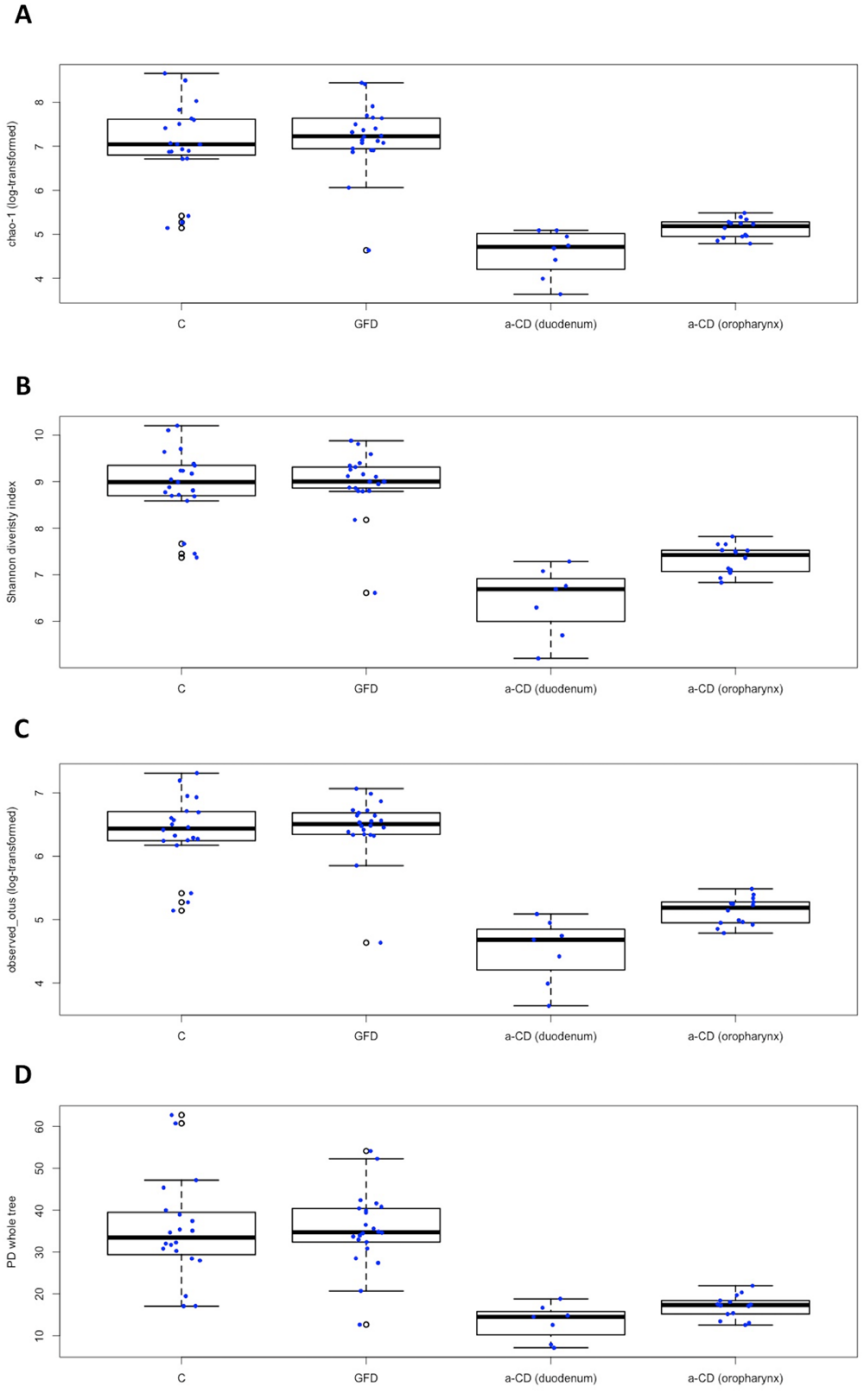
Alpha diversity of bacteria identified in oropharynx of Control (C) and gluten-free diet (GFD) groups and in oropharynx and duodenum of active celiac disease (a-CD) patients. Alpha diversity analysis was evaluated using diverse metrics in order to assess the within-sample diversity and compare the different conditions under study. (**A**) Chao-1 estimated species richness. (**B**) Shannon diversity index, (**C**) number of observed operational taxonomic units (defined by 97% identity) and (**D**) phylogenetic metric (PD_whole_tree), were calculated. Overall, the plots show a lower microbial diversity in a-CD patients and a comparable diversity between control and GFD samples.

Alpha diversity analysis was performed by using several metrics in order to assess within-sample diversity and compare the different conditions under study (Fig. 2). In particular, Chao-1 estimated species richness, Shannon diversity index, number of observed operational taxonomic units (defined by 97% identity) and phylogenetic metric (PD_whole_tree) were calculated **(**Fig. 2A–D**)**. The results highlighted a lower microbial diversity of a-CD samples versus GFD and control samples in both studied niches.

**Figure 3:**
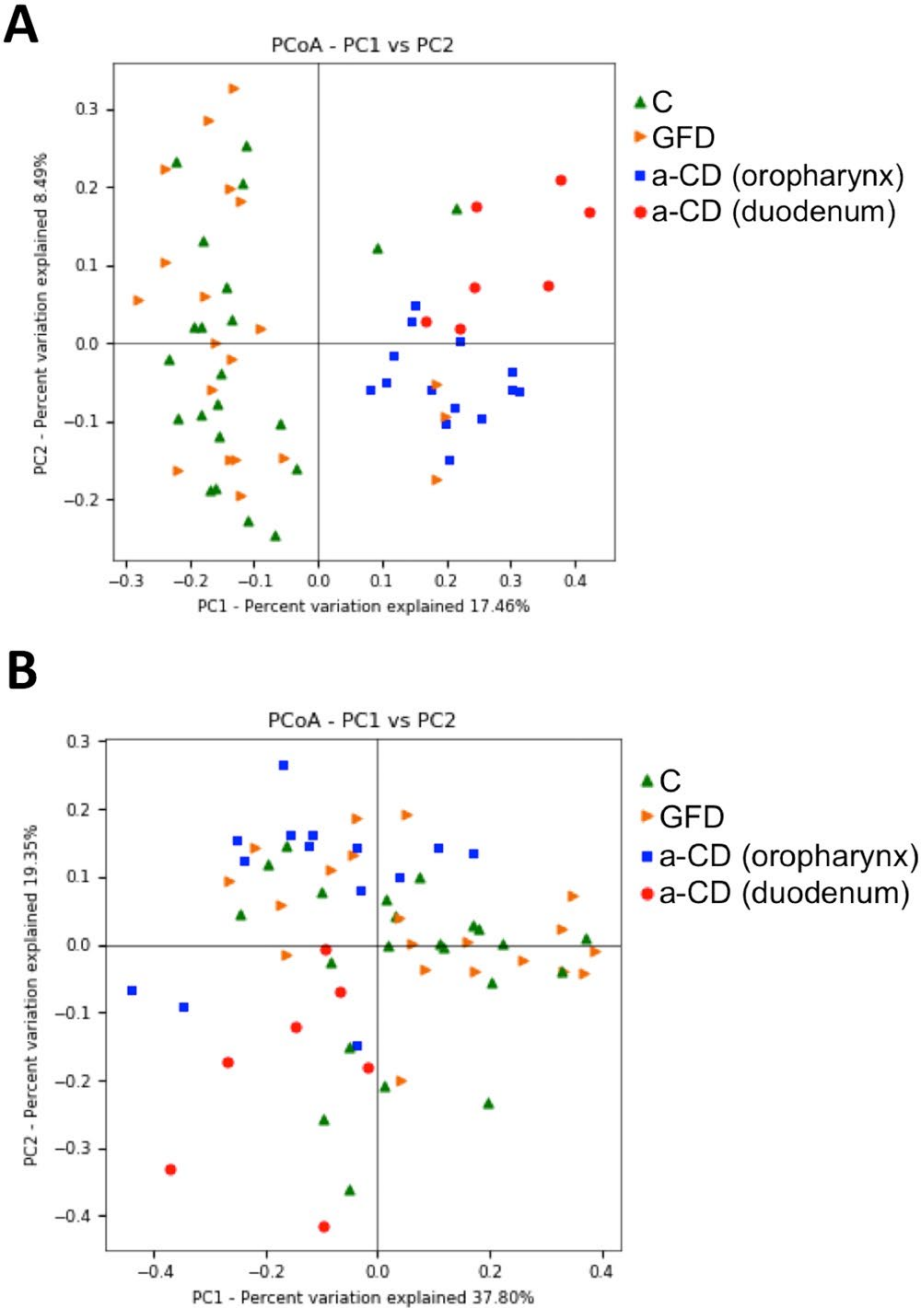
Beta diversity of bacteria identified in the Control (C), gluten-free diet (GFD) and active celiac disease (a-CD) groups. Principal coordinate analysis plots (PCoA) using the unweighted (**A**) and weighted (**B**) UniFrac distance measures. Statistical significance of groupings was assessed using the PERMANOVA test (ADONIS function) and significant results were obtained in both cases (UNWEIGHTED: P = 0.001, R2 = 0.17396; WEIGHTED: P = 0.001, R2 = 0.20489).

Principal coordinate analysis (PCoA) was performed to measure beta diversity using both unweighted and weighted UniFrac distance matrices (Fig. 3A,B). The unweighted UniFrac PCoA plots indicated a clear separation between a-CD samples and both controls and GFD along the PC1, and the cluster of celiac samples was evident. The significance of grouping samples by diagnosis categories (C, GFD and a-CD) was assessed through partitioning sums of squares (ADONIS function) of UniFrac unweighted and weighted distance matrix (UNWEIGHTED: P = 0.001, R2 = 0,17396; WEIGHTED: P = 0.001, R2 = 0,20489). Both weighted and unweighted based grouping were significant.

**Figure 4:**
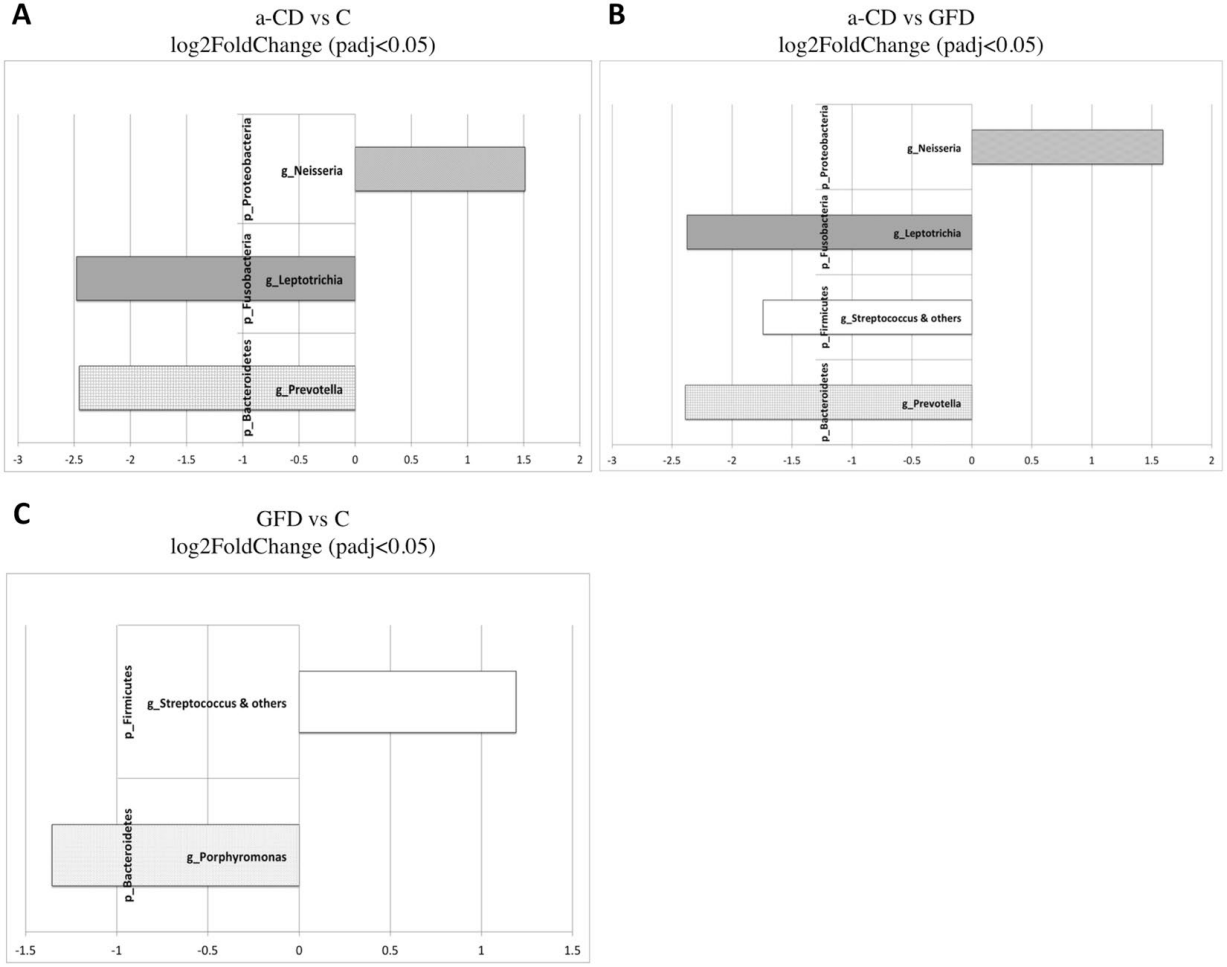
OTU differential abundance testing. Significantly differentially abundant OTUs in the three pairwise comparisons were detected using the DESeq2 extension contained in the phyloseq package. Bars represent the log2FoldChange values of significantly (FDR padj < 0.05) different taxonomic groups in a-CD vs control (**A**), a-CD vs GFD (**B**) and GFD vs control samples (**C**). Both genus and phylum levels are shown.

To identify OTUs that differed between two groups in a pairwise manner, we performed differential abundance analysis using the DESeq2 negative binomial Wald test. The abundance of the *Neisseria* genus (p-Proteobacteria) was higher in the a-CD microbiome than in either the controls (Fig. 4A) or GFD patients (Fig. 4B), whereas the abundances of *Prevotella* (p-Bacteroidetes) and *Leptotrichia* (p-Fusobacteria) were lower in the a-CD microbiome than in the GFD and in control microbiomes (Fig. 4A,B). Lastly, *Streptococcus* (p-Firmicutes) was more and less abundant, respectively, in GFD microbiomes than in the a-CD and control microbiomes (Fig. 4B,C**)**, and *Porphyromonas* (p-Bacteroidetes) was less abundant in GFD respect to C microbiomes (Fig. 4C).

### Culture based characterization of the oropharyngeal microbiota

Culture microbiological analysis of the oropharyngeal microbiota was also performed. Enriched and selective media were used to enumerate cultivable aerobic and facultative anaerobic bacteria. Relative abundance analysis (percentage) of bacteria showed cultivable species belonging to Firmicutes, Actinobacteria, Bacteroidetes and Proteobacteria phyla (Table S4). Most cultivable bacteria belonged to Firmicutes (*Streptococcus*, *Veilonella*, *Gemella*, and *Staphylococcus*) that, moreover, were less abundant in a-CD (65.01%) than in either GFD (84.80%) or controls (89.26%), with *Streptococcus* being the most abundant genus in all groups. Furthermore, *Rothia* (p-Actinobacteria) was present in controls and GFD but not in a-CD samples, whereas *Actinomyces* (p-Actinobacteria) was abundant in a-CD samples (Table S4). *Neisseria* (p-Proteobacteria) was more abundant (p < 0.001) in a-CD (13%) than in either GFD (7%) or controls (8%). The abundance of both genera *Neisseria* and *Actinomyces* in the oropharyngeal area could be due to the same metabolic relationship that occurs in dental plaque. Namely, the propionic acid produced by *Actinomyces* can be used by *Neisseria* as a growth substrate^11^.

Lastly, Bacteroidetes species were less frequent and less frequently cultured in each group. Around one-third of oral bacteria cannot be cultured using conventional methods: some bacteria have specific requirements for nutrients^12,13^. In fact, oral bacteria have evolved as part of multispecies biofilms, and thus many must interact with other bacterial species to grow^12,13^. In this study, all aerobic and facultative anaerobic species known to be cultivable were recovered. Therefore, although the culture-based characterization of microbiota is not fully comparable to the results obtained via high-throughput sequencing, this approach highlights the viability of the most prevalent cultured species, which includes those belonging to the *Neisseria* genus (Table S4).

### Prediction of the metabolic functions profiles in oropharyngeal microbial communities

**Figure 5:**
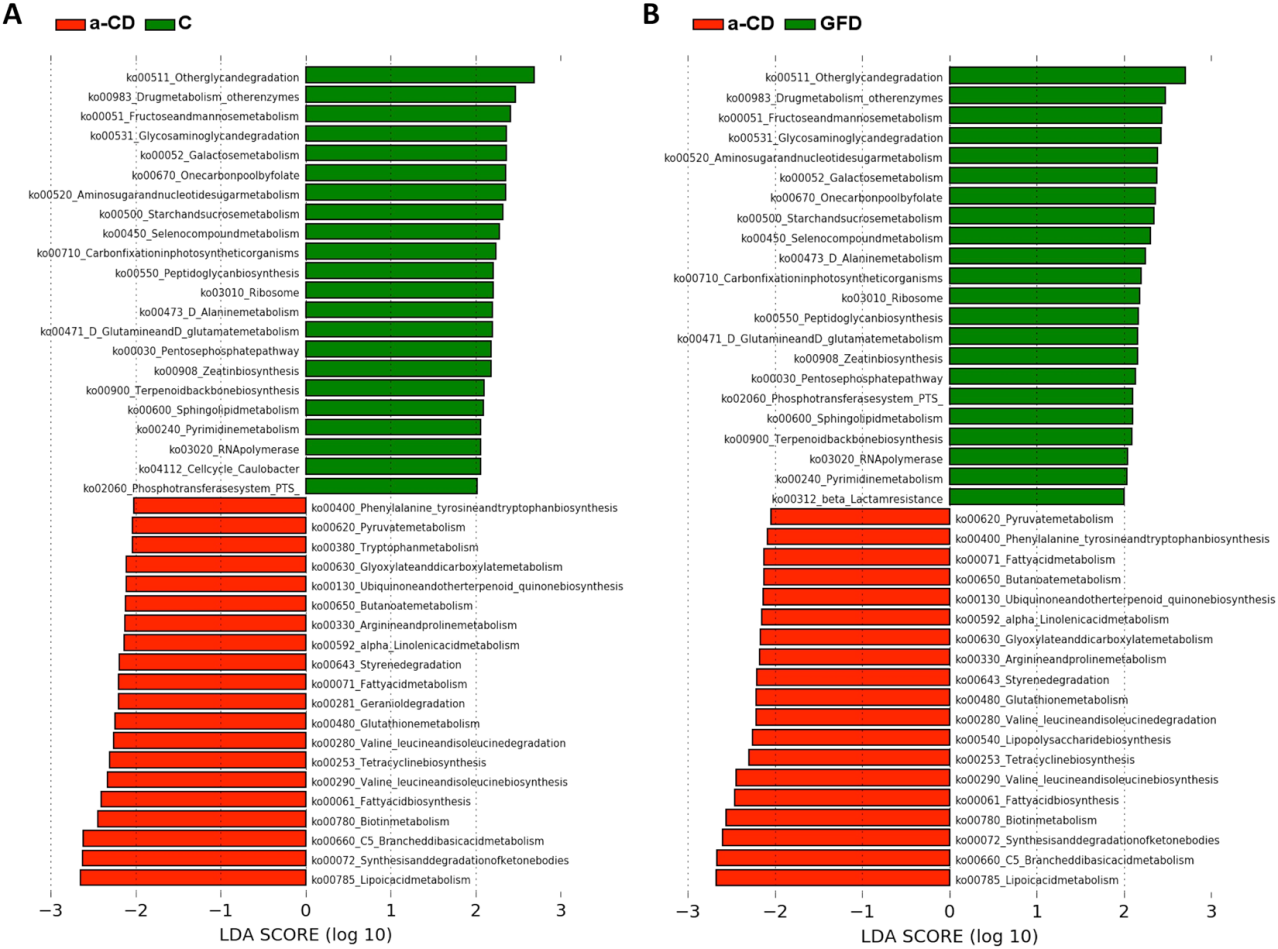
Prediction and analysis of microbiome functional profiles. The analysis of the differential abundance of gene functions associated with microbiome composition showed that the metabolic potential of a-CD microbiomes differed greatly from those of control (**A**) and GFD microbiomes (**B**). The alpha value for the factorial Kruskal-Wallis test among classes <0.05 was considered significant and the threshold on the logarithmic LDA score for discriminative features was 2.

The functional profiles in the oropharyngeal microbiomes were predicted using the PICRUSt tool. Weighted Nearest Sequenced Taxon Index (weighted NSTI) scores were calculated to assess the accuracy of the predictions for each sample (Table S5). All the values were <0.06 and, according to PICRUSt authors, who determined accuracy (Spearman) vs. NSTI, scores <0.06 are considered quite good. Metagenome predictions were categorized in KEGG pathways and differential analyses were carried out. The metabolic potential of a-CD microbiomes differed greatly from that of control (Fig. 5A) and GFD microbiomes (Fig. 5B). Indeed, the a-CD microbiome was characterized by a greater metabolic potential for degradation of amino acids, metabolism of lipid and ketone bodies and microbial antioxidant defense mechanisms, whereas genes associated with polysaccharide metabolism predominated in the control and GFD microbiomes. The distribution in the proportion of specific KEGG pathways selected by LDA score and by literature assigned to samples of the three groups are shown in Fig. S4. Boxes indicate the IQR (75th to 25th of the data). The median value is shown as a line within the box and the mean value as a star. Whiskers extend to the most extreme value within 1.5*IQR. Outliers are shown as crosses.

The contributions of bacterial orders to these selected KEGG pathways were calculated for each of the three studied groups. The gene counts per OTU per sample were weighted according to the number of genes with which each bacterial order was predicted to contribute. The weighted percentages are reported, as heat maps, in Fig. S5. The weight of the contributions was differently distributed across the orders among the three groups. In a-CD almost the entire metabolic contribution was assigned to Neisseriales, which was the most predominant order.

## Discussion

In our study we first investigated the oropharyngeal microbiome in a-CD patients and found that the Proteobacteria was the most abundant phylum, with *Neisseria* being the most abundant genus. Particularly, we highlighted in the oropharynx of a-CD patients a microbial imbalance similar to that we previously described in a-CD duodenal microbiome^3^. Further, we observed an almost coincident oropharyngeal microbiome in controls and GFD subjects. In fact, in both microbiomes the Bacteroidetes dominated and the *Prevotella* genus was abundant. This latter result, in agreement with previously reported oral microbial profiles in healthy subjects^14,15^, supports the oropharyngeal microbiome restoration after a GFD.

Our findings raised a series of questions, to which we will try to give possible answers based also on the findings to date known in literature.

What trigger the abundance of *Neisserial* species in the a-CD oropharyngeal microbiome and what does it involve? About the hypothetical factors triggering the *Neisserial* microbial imbalance in a-CD patients, we think unlikely that the main predisposing genetic factor in CD, i.e. HLA-genetics, could impact on microbiome, at least not alone. That is why the oropharyngeal microbiome in our CD patients, all of them positive for HLA-DQ2 or-DQ8 allele presence, restored after GFD. Alternatively, we can hypothesize that antibiotic treatment and/or other conditions (i.e. viral infections), could promote oral dysbiosis. This, in turn, could lead to ectopic colonization by oral-derived bacteria of the duodenum in CD patients able in inducing gut inflammation and activation of the immune system. To support this hypothetical mechanism *Klebsiella pneumonia* (Kp-2H7) strain resistant to multiple antibiotics, isolated from salivary microbiota of patients with Crohn’s disease, was recently demonstrated to colonize the gut and to induce chronic intestinal inflammation in germ-free mice^16^. In line with the aforementioned results, we previously demonstrated that *Neisseria flavescens* strains isolated from duodenal and from oral microbiota, were able to induce inflammation in dendritic cells and in *ex-vivo* duodenal mucosal explants of healthy controls^3^. Concerning the role of viral infections, reovirus infection (Type 1 Lang), an avirulent pathogen, was recently demonstrated to perturb intestinal immune homeostasis and trigger CD by promoting Th1 immunity to dietary antigen^5^.

Can the metabolic changes predicted in association with the a-CD microbiome make sense in the context of the disease? The predicted function modules indicated that a-CD microbiome have higher metabolic potential for the degradation of amino acids and energy production from lipids and ketone bodies than control and GFD microbiomes. The latter metabolic potential could likely be beneficial for the Neisseria that could take advantage from the intestinal epithelium exfoliation occurring in the early stage of CD. An increased use of ketone bodies as a source of energy in a-CD patients has been previously reported^17^, and our data suggest that this metabolic imbalance could, in part, be mediated by *Neisseria* abundance. Further, the predicted a-CD metabolic profiles were characterized by enhanced protective systems, which bacteria, fungi and parasites use to escape the host oxidative defense during the immune response against infectious processes and reported to be involved in bacterial virulence^18^. Considering the previously described inflammatory effect of the *Neisseria* strains^3^, we may hypothesize that the increased bacterial defense mechanisms to which *Neisseria* is predicted to largely contribute, may be enhanced to overcome the host immune response in a-CD patients. Most studies of the genome-derived metabolic signature of *Neisseria* investigated the invasive behavior in commensal pathogens^19^. They reported that the expression of genes involved in stress responses (i.e. glutathione metabolism) and aminoacid metabolism generally differ between hyperinvasive and carriage lineages, but energy production, lipid transport and metabolism pathways also differed^19^.

Does the a-CD associated microbial imbalance precede or follow the onset of the disease? If the increase of *Neisserial* species occurs early or late in the natural history of CD, in our opinion, can be clarified only by monitoring the oropharyngeal microbiome changes in at-risk for CD groups, such as potential CDs (PCDs), from diagnosis to the overt disease, which occurs in about one third of the patients^20,21^. We have recently undertaken this study and hopefully could have in a next future further data to clarify this point.

Finally, what is the relationship between gluten and the a-CD associated dysbiosis, in oropharynx and duodenum? First of all, the restoration of oropharyngeal microbiome after GFD, suggests that alone or in the presence of host genetic and non-genetic factors, gluten contributes to trigger or at least to exacerbate dysbiosis. Regarding the oral microbiota, we found that *Rothia* and *Prevotella*, which are strains previously reported to be active in degrading gluten^22–24^, were lower in a-CD than in GFD and controls, suggesting a reduced gluten-degrading capacity in a-CD microbiome. In the mucosal duodenal epithelium, we previously showed *Neisseria* spp enter early endocytic vesicles^3^, that are also the endocytic route of gliadin peptides^25^, so probably interfering in gluten degradation and contributing to promote cell stress/innate immune activation^25^.

In conclusion, our results of the increased presence of *Neisseria* strains in a-CD oropharyngeal microbiome suggest a continuum of a-CD microbial composition from mouth to duodenum. We may speculate that microbiome characterization in the oropharynx, which is a less invasive sampling than the duodenum, could contribute to investigate the role of dysbiosis in CD pathogenesis.

## Methods

### Study groups and samples

Fifty-six Caucasian individuals were recruited over a two years period among patients attending the Departments of Gastroenterology of the Universities of Salerno and of Roma-Tor Vergata, and the Ambulatory of Molecular Medicine and Medical Biotechnologies at the University Federico II, of Naples, Italy. All enrolled subjects did not present evident signs of oral inflammation (i.e. dental caries, bloody or sore gums). Exclusion criteria for enrolment included treatment with antibiotics, proton pump inhibitors and antiviral or corticosteroid assumption in the two months before sampling. Study groups: (1) 14 individuals on a gluten-containing diet with CD-like symptoms and positive for CD-specific antibodies (IgA anti-endomysium and/or anti-tissue transglutaminase), in whom CD was subsequently confirmed by mucosal villous atrophy of duodenum biopsies (Marsh-Oberhuber classification IIIa to IIIc); (2) 22 CD patients on a GFD for at least 2 years; and (3) 20 controls, (both GFD patients and the healthy controls were negative for CD-specific antibodies). We collected blood samples from all participants (to obtain DNA and serum) and two oropharyngeal swabs (Eswab^TM^ Copan, Murrieta, CA, USA), touching the back wall of the oropharynx and no other oral structures, in a Liquid Amies Elution Swab (Eswab) collection and transport system for microbiological assays. We chose to study oropharynx microbiome in order to obtain bacteria adherent to epithelial layer, and therefore this sample could be more representative of the gastrointestinal tract. The swabs were immediately cooled with 10% glycerol in dry ice and stored at −80 °C for genetic and microbiological analysis. In 7/14 a-CD patients a duodenal biopsy (available after histology) was used for microbiome characterization, cooled in dry ice and stored at −80 °C until analysis. All subjects gave their written informed consent to participate in the study that was carried on according to the tenets of the Helsinki Declaration and approved by the University of Naples Federico II Ethics Committee (Prot. N. 36/13).

### DNA extraction and 16S rRNA sequencing

Total genomic DNA was extracted by phenol-chloroform method. DNA quantity and quality were evaluated with the NanoDrop® ND-1000 UV-Vis spectrophotometer (NanoDrop Technologies, Wilmington, DE, USA) and 0.8% agarose gel. All extractions were performed in a pre-PCR designated room. Amplification of the V4-V6 regions of the 16S rRNA gene was performed following the Illumina 16S Metagenomic Sequencing Library Preparation workflow^25,26^. The Illumina overhang adapter sequences were added to V4-V6 locus‐specific primers (Forward primer: CAGCAGCCGCGGTAATAC and Reverse primer: TGACGACAGCCATGC). The first PCR reaction was performed with the 2.5 × 5 PRIME MasterMix (Eppendorf) using the following cycling conditions: 94 °C for 2 min, 94 °C for 40 s, 60 °C for 40 s and 65 °C for 40 s for 40 cycles, 65 °C for 10 min. DNA fragments were then analyzed on 2% agarose gel and purified through Agencourt AMPure XP Beads (Beckman Coulter, Brea, CA, USA). The Illumina indices were inserted on each amplified region according to the Nextera XT protocol (Illumina, San Diego, CA, USA). The V4-V6 amplified regions of each sample were purified and quality-assessed using the 2100 Bioanalyzer Instrument (Agilent, Santa Clara, CA, USA). Library quantification was performed using the Qubit dsDNA BR assay kit (Life Technologies, CA, USA). Up to 38 libraries were pooled and combined to 25% of PhiX control for sequencing using a MiSeq v3 reagent cartridge. Thus, 2 sequencing runs were totally performed with the Illumina MiSeq System (PE 300 × 2). Before sequencing reaction, all the DNA libraries were quantified at Qubit by picogreen assay in order to obtain a pool of equimolar libraries, so ensuring a normalization across the different samples sequenced in the same run.

### Bioinformatics analysis

Raw sequences were quality-filtered and processed using QIIME v1.9.1^27^. Chimeras were removed using usearch61^28^. Chimera-filtered sequences were assigned to operational taxonomic units (OTUs) using an open-reference OTU picking approach, with usearch61 at a 97% identity. For each OTU, reads with the highest frequencies were chosen as representative sequences. Representative OTUs were assigned to different taxonomic levels (from phylum to genus) using the bacterial Greengenes v.13_8 dataset^29^. Normalization of OTUs table was performed through metagenomeSeq’s CSS (cumulative sum scaling) transformation^30^. For DESeq please see^31^. Alpha diversity analysis was performed through several metrics in order to assess the within-sample diversity and compare the different conditions under study. In particular, Shannon diversity index, Chao-1 estimated species richness, phylogenetic metric (PD_whole_tree) and number of observed operational taxonomic units (defined by 97% identity) were calculated.

UniFrac distance matrices were generated from OTU assignment and used to create principal coordinates analysis (PCoA) plots^32^. To evaluate whether the grouping of samples by diagnosis categories (C, GFD and a-CD) was statistically significant we used ADONIS function, which partitions the sums of squares of distance matrix derived from unweighted and weighted UniFrac measurements. The Phyloseq R package^33^ was used to exploit the DESeq2 official extension and to perform hierarchical clustering analysis using the unweighted UniFrac distance and the Ward’s method^34^. Microbial functional contents from 16S rRNA were predicted through Phylogenetic Investigation of Communities by Reconstruction of Unobserved States (PICRUSt)^35^. The contribution of OTUs to some predicted functions was extracted by PICRUSt and weighted by the number of genes to which each OTU contributes. The functions were chosen based on data about their involvement in celiac disease. The metagenomic matrix (in biom format), was then used as input in HUMAnN (The HMP Unified Metabolic Analysis Network) software^36,37^ to generate gene and pathway summaries. HUMAnN outputs were used for differential abundance analysis with Linear discriminant analysis effect size evaluation (LefSe) (http://huttenhower.sph.harvard.edu/galaxy/) in order to identify gene families that differed consistently between sample groups. Box plots and statistics of the distribution in the proportion of specific KEGG pathways were computed using Statistical Analysis of Metagenomic Profiles (STAMP)^38^.

### Microbiological analysis

Culture-dependent microbiological analysis of aerobic and facultative anaerobic species present in oropharyngeal swabs was performed for all enrolled individuals by cfu method. Enriched and differential media were used to analyze all oropharyngeal swabs collected: Becton Dickinson (BD) Trypticase Soy agar with 5% sheep blood, McConkey agar and BD Sabouraud agar were used in aerobic condition, while BD Trypticase Soy agar with 5% sheep blood in anaerobic environment. Standard microbiological culture was followed by mass spectrometry identification of bacterial isolates using the Matrix Assisted Laser Desorption/Ionization (MALDI) mass spectrometer (Bruker Daltonics MALDI Biotyper, Fremiont, CA, USA; VITEK MS system, bioMérieux S.A. Marcy l’Etoile, France)^39,40^.

### Statistical analysis

Comparison of the general characteristics among the three study groups was performed with the Fisher’s test. Differences in abundance of specific taxa among the three study groups were estimated using the non-parametric Kruskal-Wallis test. Differences in operational taxonomic units were considered statistically significant at p ≤ 0.05. In a post-hoc analysis we applied the Dunn’s test to the taxa abundances that were significantly different, to determine which levels of the independent variable differ from each other level. The p-value was corrected for multiple testing using the Benjamini-Hochberg approach.

Pearson correlation between microbiome, age, GFD period and gender annotation was calculated through R function feature.assoc () included in swamp package (https://CRAN.R-ptoject.org/package=swamp). The statistical significance of sample groupings was calculated by the Permutational Multivariate Analysis Of Variance through ADONIS function.

The negative binomial Wald test by DESeq2 was used to perform differential abundance analysis across two sample categories. Linear discriminant analysis (LDA) Effect Size (LEfSe tool) was applied to identify microbial differences between groups in terms of microbiome-associated gene functions. Alpha value for the factorial Kruskal-Wallis test among classes < 0.05 was considered significant and the threshold on the logarithmic LDA score for discriminative features was 2. Differences in culture-dependent data between groups were analyzed by test U of Mann-Whitney, using SPSS software, IBM SPSS Statistics. A P value < 0.05 indicated a statistically significant difference.

### Availability of data

The datasets generated and analyzed during the current study have been submitted to the NCBI SRA repository and will be available after paper acceptance.

## Electronic supplementary material

Supplemental material
