# Supplementary material for: Oropharyngeal microbiome evaluation highlights Neisseria abundance in active celiac patients
Source: Sci Rep. 2018 Jul 23;8:11047. doi: 10.1038/s41598-018-29443-1 (PMC6056421; doi:10.1038/s41598-018-29443-1)
Supplement: Supplementary file 1 — Supplemental material [file 41598_2018_29443_MOESM1_ESM.docx]

Supplementary material for

Oropharyngeal microbiome evaluation highlights *Neisseria* abundance in active celiac patients

Laura Iaffaldano^1,*^, Ilaria Granata^2,1*^, Chiara Pagliuca^1,3,4^, Maria Valeria Esposito^1,3,4^, Giorgio Casaburi^5,9^, Giuliana Salerno^3^, Roberta Colicchio^3,4^, Marina Piccirillo^2,6^, Carolina Ciacci^7^, Giovanna Del Vecchio Blanco^8^, Mario Rosario Guarracino^2^, Paola Salvatore^1,3,4^, Francesco Salvatore^1,4^, Valeria D’Argenio^1,3,4,**^ and Lucia Sacchetti^1,4**^.

^1^Ceinge Biotecnologie Avanzate scarl, Naples, Italy; ^2^LabGTP (Laboratory of Genomics, Transcriptomics and Proteomics), Institute for High Performance Computing and Networking (ICAR), National Research Council (CNR), Naples, Italy; ^3^Department of Molecular Medicine and Medical Biotechnologies, University of Naples Federico II, Naples, Italy; ^4^Task Force on Microbiome Studies, University of Naples Federico II, Naples and Ceinge Biotecnologie Avanzate scarl, Naples, Italy; ^5^Department of Microbiology and Cell Science, University of Florida, Space Life Science Lab, Merritt Island, FL, USA; ^6^Department of Environmental, Biological and Pharmaceutical Sciences and Technologies, University of Campania Luigi Vanvitelli; ^7^Department of Medicine and Surgery, University of Salerno, Salerno, Italy; ^8^Department of System Medicine, University of Rome Tor Vergata, Rome, Italy.

^9^Present address: Evolve Biosystems, Inc. Davis, CA 95618, USA

^*^These authors contributed equally to this work.

^**^Corresponding authors with equal contribution and responsibilities: **Lucia Sacchetti**, PhD and **Valeria D’Argenio**, MD, PhD, CEINGE-Biotecnologie Avanzate, via Gaetano Salvatore 486, Naples 80131, Italy. E-mail: sacchett@unina.it and dargenio@ceinge.unina.it.

**Table S1.** General features of the three study groups

|  | **C** | **GFD** | **a-CD** |
| --- | --- | --- | --- |
| **No. of subjects** | 20 | 22 | 14 |
| **Age at enrolment (years)**  **(Mean±sem.)** | 30±.4 | 32±.4 | 26±.4 |
| **Gender (% Females)** | 70% | 81%* | 57%* |
| **Vaginal Delivery** | 70% | 82% | 71% |
| **Breastfeeding** | 70% | 86% | 71% |
| **Presence of CD antibodies**  **(EMA and/or TG2 IgA)** | 0(+)/20 | 0(+)/22 | 14(+)/14 |
|  |  |  |  |

a-CD, active celiac disease patients; GFD, gluten-free diet celiac patients, C, healthy controls, EMA, anti-endomysium, TG2, anti-tissue transglutaminase.

* Statistically significant difference at Fisher's test between GFD and a-CD (p=0.001)

**Table S2.** Samples metadata obtained by 16S rRNA sequencing of the oropharyngeal and duodenal microbiomes in the study groups (QIIME tool).

|  | **Total number of input sequences** | | | | **Quality filtered sequences** | | | | **Counts/sample** | | | |
| --- | --- | --- | --- | --- | --- | --- | --- | --- | --- | --- | --- | --- |
|  | **C** | **GFD** | **a-CD (oropharynx)** | **a-CD (duodenum)** | **C** | **GFD** | **a-CD (oropharynx)** | **a-CD (duodenum)** | **C** | **GFD** | **a-CD (oropharynx)** | **a-CD (duodenum)** |
| **Mean** | 22540,95 | 33626,05 | 900,14 | 2112,71 | 22293,30 | 32290,77 | 878,57 | 2112,71 | 17102,75 | 22141,32 | 707,07 | 522,57 |
| **SEM** | 2718,94 | 5811,31 | 50,36 | 491,84 | 2712,12 | 5548,75 | 52,77 | 491,84 | 1946,76 | 2239,15 | 51,64 | 89,55 |
|  | **C** | | | **GFD** | | | **a-CD (oropharynx)** | | | **a-CD (duodenum)** | | |
| **Non-filtered** | 11 phyla | | | 12 phyla | | | 6 phyla | | | 6 phyla | | |
|  | 21 classes | | | 22 classes | | | 13 classes | | | 12 classes | | |
|  | 30 orders | | | 33 orders | | | 21 orders | | | 21 orders | | |
|  | 62 families | | | 68 families | | | 37 families | | | 42 families | | |
|  | 120 genera | | | 133 genera | | | 66 genera | | | 66 genera | | |
|  |  | | |  | | |  | | |  | | |
| **mean > 1%** | 4 phyla | | | 5 phyla | | | 4 phyla | | | 5 phyla | | |
|  | 7 classes | | | 7 classes | | | 7 classes | | | 8 classes | | |
|  | 7 orders | | | 9 orders | | | 8 orders | | | 11 orders | | |
|  | 12 families | | | 13 families | | | 9 families | | | 13 families | | |
|  | 12 genera | | | 11 genera | | | 10 genera | | | 14 genera | | |

| **Table S3.** Dunn's test performed on significant differentially abundant taxa according to Kruskal-Wallis test | | | | | | | | | | | | | |  |  |  |  |  | |  |
| --- | --- | --- | --- | --- | --- | --- | --- | --- | --- | --- | --- | --- | --- | --- | --- | --- | --- | --- | --- | --- |
|  |  |  |  |  |  |  |  |  |  |  |  |  | |  |  |  |  |  | |  |
| **PHYLUM** |  |  |  | **CLASS** |  |  |  | **ORDER** |  |  |  | **FAMILY** | |  |  |  | **GENUS** |  | |  |
| Firmicutes* |  |  |  | Flavobacteriia* | |  |  | Flavobacteriales* | |  |  | Lachnospiraceae* | | |  |  | (Bacteroidetes);Porphyromonas* | | | |
|  | C | GFD |  |  | C | GFD |  |  | C | GFD |  |  | C | | GFD |  |  | C | GFD | |
| GFD | 0,1674 |  |  | GFD | 0,0746 |  |  | GFD | 0,0746 |  |  | GFD | 0,1659 | |  |  | GFD | 0,0328* |  | |
| a-CD | 0.0223* | 0.0030* |  | a-CD | 0,0927 | 0.0061* |  | a-CD | 0,0927 | 0.0061* |  | a-CD | 0.0144* | | 0.0016* |  | a-CD | 0,4969 | 0,0292* | |
|  |  |  |  |  |  |  |  |  |  |  |  |  |  | |  |  |  |  |  | |
| Bacteroidetes* |  |  |  | Clostridia* |  |  |  | Clostridiales* | |  |  | Porphyromonadaceae* | | |  |  | (Fusobacteria);Leptotrichia* | | | |
|  | C | GFD |  |  | C | GFD |  |  | C | GFD |  |  | C | | GFD |  |  | C | GFD | |
| GFD | 0,2374 |  |  | GFD | 0,4342 |  |  | GFD | 0,4342 |  |  | GFD | 0.0161* | |  |  | GFD | 0,4809 |  | |
| a-CD | 0.0008* | 0.0028* |  | a-CD | 0.0021* | 0.0014* |  | a-CD | 0.0021* | 0.0014* |  | a-CD | 0,2892 | | 0,0615 |  | a-CD | 0.0027* | 0.0038* | |
|  |  |  |  |  |  |  |  |  |  |  |  |  |  | |  |  |  |  |  | |
| Proteobacteria* |  |  |  | Bacteroidia * | |  |  | Bacteroidales* | |  |  | [Paraprevotellaceae]* | | |  |  | (Bacteroidetes);[Prevotella]* | | | |
|  | C | GFD |  |  | C | GFD |  |  | C | GFD |  |  | C | | GFD |  |  | C | GFD | |
| GFD | 0,4238 |  |  | GFD | 0,261 |  |  | GFD | 0,261 |  |  | GFD | 0.0032* | |  |  | GFD | 0.0032* |  | |
| a-CD | 0.0012* | 0.0009* |  | a-CD | 0.0007* | 0.0022* |  | a-CD | 0.0007* | 0.0022* |  | a-CD | 0,0627 | | 0,1549 |  | a-CD | 0,0627 | 0,1549 | |
|  |  |  |  |  |  |  |  |  |  |  |  |  |  | |  |  |  |  |  | |
|  |  |  |  | Betaproteobacteria* | |  |  | Neisseriales* | |  |  | Leptotrichiaceae* | | |  |  | (Proteobacteria);Neisseria* | | | |
|  |  |  |  |  | C | GFD |  |  | C | GFD |  |  | C | | GFD |  |  | C | GFD | |
|  |  |  |  | GFD | 0,3017 |  |  | GFD | 0,2957 |  |  | GFD | 0,4008 | |  |  | GFD | 0,3117 |  | |
|  |  |  |  | a-CD | 0.0003* | 0.0001* |  | a-CD | 0.0005* | 0.0001* |  | a-CD | 0.0148* | | 0.0138* |  | a-CD | 0.0006* | 0.0002* | |
|  |  |  |  |  |  |  |  |  |  |  |  |  |  | |  |  |  |  |  | |
|  |  |  |  |  |  |  |  |  |  |  |  | Veillonellaceae* | | |  |  | (Bacteroidetes);Prevotella* | | | |
|  |  |  |  |  |  |  |  |  |  |  |  |  | C | | GFD |  |  | C | GFD | |
|  |  |  |  |  |  |  |  |  |  |  |  | GFD | 0,0971 | |  |  | GFD | 0,4048 |  | |
|  |  |  |  |  |  |  |  |  |  |  |  | a-CD | 0.0183* | | 0,1181 |  | a-CD | 0.0017* | 0.0014* | |
|  |  |  |  |  |  |  |  |  |  |  |  |  |  | |  |  |  |  |  | |
| alpha = 0.05 |  |  |  |  |  |  |  |  |  |  |  | Prevotellaceae* | | |  |  |  |  |  | |
| Reject Ho if p <= alpha/2 | |  |  |  |  |  |  |  |  |  |  |  | C | | GFD |  |  |  |  | |
| * significant |  |  |  |  |  |  |  |  |  |  |  | GFD | 0,4048 | |  |  |  |  |  | |
|  |  |  |  |  |  |  |  |  |  |  |  | a-CD | 0.0017* | | 0.0014* |  |  |  |  | |
|  |  |  |  |  |  |  |  |  |  |  |  |  |  | |  |  |  |  |  | |
|  |  |  |  |  |  |  |  |  |  |  |  | Neisseriaceae* | | |  |  |  |  |  | |
|  |  |  |  |  |  |  |  |  |  |  |  |  | C | | GFD |  |  |  |  | |
|  |  |  |  |  |  |  |  |  |  |  |  | GFD | 0,2957 | |  |  |  |  |  | |
|  |  |  |  |  |  |  |  |  |  |  |  | a-CD | 0.0005* | | 0.0001* |  |  |  |  | |

| **Table S4.** Culture-dependent microbiological analysis of the oropharyngeal  microbiota in the three study groups. The table shows mean percentage  values of bacterial species present in the samples with a percentage  greater than 3% | | | | | | | | | | | | | |  |  |
| --- | --- | --- | --- | --- | --- | --- | --- | --- | --- | --- | --- | --- | --- | --- | --- |
|  | **C** | **GFD** | **a-CD** |  |  | **C**  **(N= 20)** |  | | | **GFD**  **(N= 22)** |  | **a-CD**  **(N= 14)** |  | | |
| **Phylum** | **Mean percentage of cfu^a^** | | | **Genus** |  | **Mean percentage of cfu** | | |  | **Mean percentage of cfu** |  | **Mean percentage of cfu^a^** |  | | |
| *Firmicutes* | *89.26*% | *84.80*% | *65.01*% | ***Staphylococcus*** |  | *0%* | |  | | *0%* |  | *<3%* |  | |  |
|  |  |  |  | *(S. aureus; S. epidermidis; S. capitis)* |  |  | |  | |  |  |  |  | |  |
|  |  |  |  | ***Streptococcus*** |  | *79.48%* | |  | | *69.64%* |  | *55.33%^**^* |  | |  |
|  |  |  |  | *(Beta hemolitic, Non beta hemolitic, Salivarius, Mitis, Sanguinis, Anginosus groups)^b^* |  |  | |  | |  |  |  |  | |  |
|  |  |  |  | ***Veilonella*** |  | *9.27%* | |  | | *13.45%* |  | *7.58%^***^* |  | |  |
|  |  |  |  | *(V. atypica, V. dispar, V. parvula)* |  |  | |  | |  |  |  |  | |  |
|  |  |  |  | ***Gemella*** |  | *<3%* | |  | | *<3%* |  | *0%* |  | |  |
|  |  |  |  | *(G. haemolysans, G. sanguis)* |  |  | |  | |  |  |  |  | |  |
|  |  |  |  |  |  |  | |  | |  |  |  |  | |  |
| *Proteobacteria* | *8.04*% | *7.01*% | *13*% | ***Neisseria*** |  | *8.04%* | |  | | *7.01%* |  | *13%^***^* |  | |  |
|  |  |  |  | *(N. flavescens, N. perflava, N. subflava, N. macacae, N. cinerea, N. mucosa)* |  |  | |  | |  |  |  |  | |  |
|  |  |  |  |  |  |  | |  | |  |  |  |  | |  |
| *Actinobacteria* | *<3%* | *7.37*% | *19*% | ***Actinomyces*** |  | *<3%* | |  | | *0%* |  | *19%^***^* |  | |  |
|  |  |  |  | *(A. oris)* |  |  | |  | |  |  |  |  | |  |
|  |  |  |  | ***Rothia*** |  | *<3%* | |  | | *7.37%^*^* |  | *0%^***^* |  | |  |
|  |  |  |  | *(R. dentocariosa, R. mucilaginosa)* |  |  | |  | |  |  |  |  | |  |
|  |  |  |  |  |  |  | |  | |  |  |  |  | |  |
| *Bacteroidetes* | *<3%* | *<3%* | *<3%* | ***Prevotella*** |  | *<3%* | |  | | *<3%* |  | *<3%* |  | |  |
|  |  |  |  | *(P. melaninogenica, P. pallens)* |  |  | |  | |  |  |  |  | |  |
|  |  |  |  |  |  |  | |  | |  |  |  |  | |  |

**Table S5**. Weighted Nearest Sequenced Taxon Index (weighted NSTI) scores calculated to assess the accuracy of the predictions of metabolic functions for each sample. Scores <0.06 were considered quite good according to PICRUST authors.

| **Sample** | **Metric** | **Value** |
| --- | --- | --- |
| a-CD1 | Weighted NSTI | 0,012580945 |
| a-CD10 | Weighted NSTI | 0,016384274 |
| a-CD11 | Weighted NSTI | 0,014986393 |
| a-CD12 | Weighted NSTI | 0,01078625 |
| a-CD13 | Weighted NSTI | 0,013232111 |
| a-CD14 | Weighted NSTI | 0,013400819 |
| a-CD2 | Weighted NSTI | 0,020799626 |
| a-CD3 | Weighted NSTI | 0,029018026 |
| a-CD4 | Weighted NSTI | 0,031600731 |
| a-CD5 | Weighted NSTI | 0,026118534 |
| a-CD6 | Weighted NSTI | 0,029699847 |
| a-CD7 | Weighted NSTI | 0,021331981 |
| a-CD8 | Weighted NSTI | 0,020682505 |
| a-CD9 | Weighted NSTI | 0,029161615 |
| C12 | Weighted NSTI | 0,060187433 |
| C13 | Weighted NSTI | 0,025683743 |
| C18 | Weighted NSTI | 0,046369058 |
| C19 | Weighted NSTI | 0,033891333 |
| C21 | Weighted NSTI | 0,020675016 |
| C23 | Weighted NSTI | 0,02800352 |
| C24 | Weighted NSTI | 0,01661228 |
| C28 | Weighted NSTI | 0,038301714 |
| C3 | Weighted NSTI | 0,026084003 |
| C33 | Weighted NSTI | 0,032385844 |
| C35 | Weighted NSTI | 0,052589407 |
| C36 | Weighted NSTI | 0,046387692 |
| C37 | Weighted NSTI | 0,025169565 |
| C38 | Weighted NSTI | 0,03470109 |
| C39 | Weighted NSTI | 0,035919978 |
| C4 | Weighted NSTI | 0,025342704 |
| C40 | Weighted NSTI | 0,025664494 |
| C45 | Weighted NSTI | 0,019767314 |
| C46 | Weighted NSTI | 0,023842111 |
| C47 | Weighted NSTI | 0,048251057 |
| GFD1 | Weighted NSTI | 0,023187282 |
| GFD10 | Weighted NSTI | 0,034222764 |
| GFD11 | Weighted NSTI | 0,038112158 |
| GFD14 | Weighted NSTI | 0,044291598 |
| GFD15 | Weighted NSTI | 0,023232283 |
| GFD16 | Weighted NSTI | 0,029189991 |
| GFD17 | Weighted NSTI | 0,030034676 |
| GFD2 | Weighted NSTI | 0,013190687 |
| GFD20 | Weighted NSTI | 0,035874626 |
| GFD22 | Weighted NSTI | 0,039092414 |
| GFD25 | Weighted NSTI | 0,019452411 |
| GFD26 | Weighted NSTI | 0,027084927 |
| GFD27 | Weighted NSTI | 0,03244521 |
| GFD30 | Weighted NSTI | 0,036525785 |
| GFD31 | Weighted NSTI | 0,025279085 |
| GFD32 | Weighted NSTI | 0,026762128 |
| GFD34 | Weighted NSTI | 0,026205371 |
| GFD36 | Weighted NSTI | 0,025346289 |
| GFD5 | Weighted NSTI | 0,019484701 |
| GFD6 | Weighted NSTI | 0,034873515 |
| GFD8 | Weighted NSTI | 0,015952671 |
| GFD9 | Weighted NSTI | 0,026577959 |


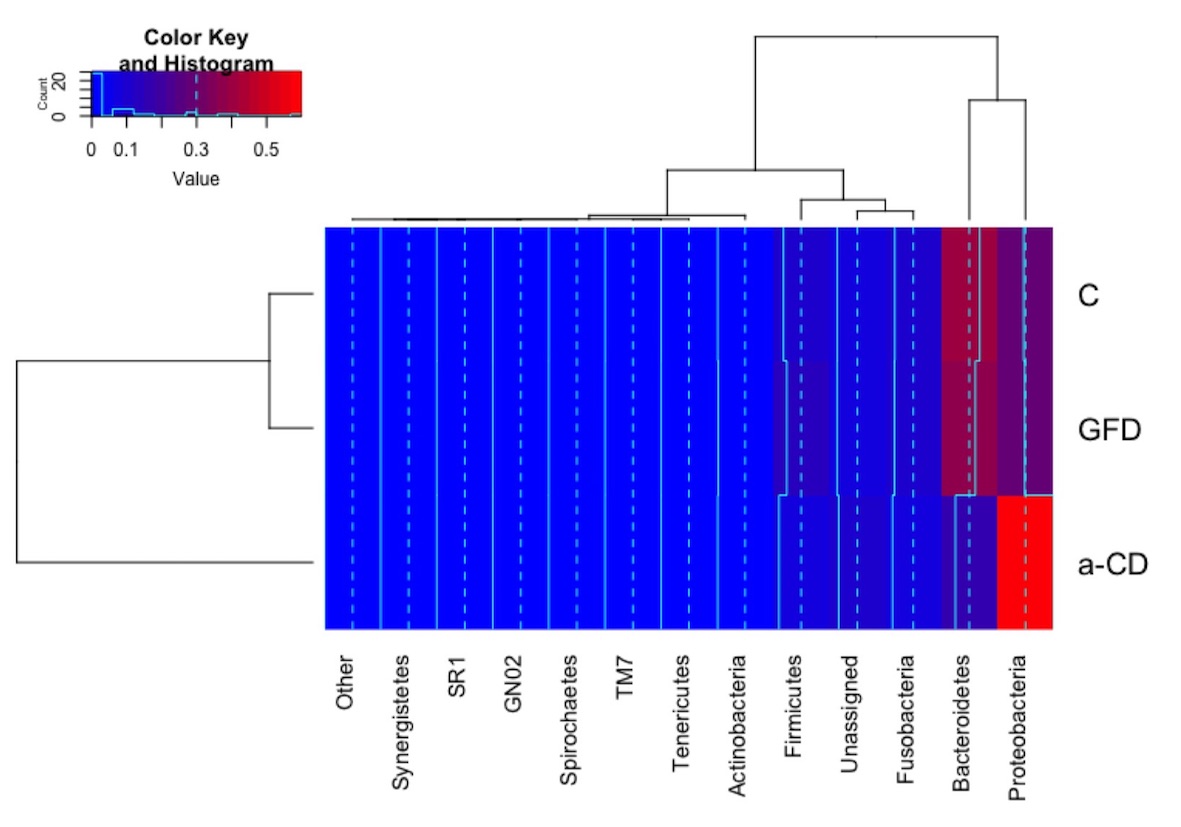


**Figure S1. Hierarchical clustering and composition analysis of oropharyngeal microbiomes in the Control (C), gluten-free diet (GFD) and active celiac disease (a-CD) groups.** Heat map generated by using gplots R package of taxa relative abundances at phylum level. All detected phyla are shown, “Other” indicates the phyla present at <0.1% frequency in the three groups. Rows and columns represented study groups and phyla, respectively. Both samples and phyla were subjected to unsupervised hierarchical clustering and the results are depicted in the dendrogram at the left and top of the figure. Similarities between the control and GFD groups determined their grouping in a single cluster, far from a-CD samples; the separation was mainly due to differences in Proteobacteria and Bacteroidetes phyla. Colors and histograms represent the abundances.


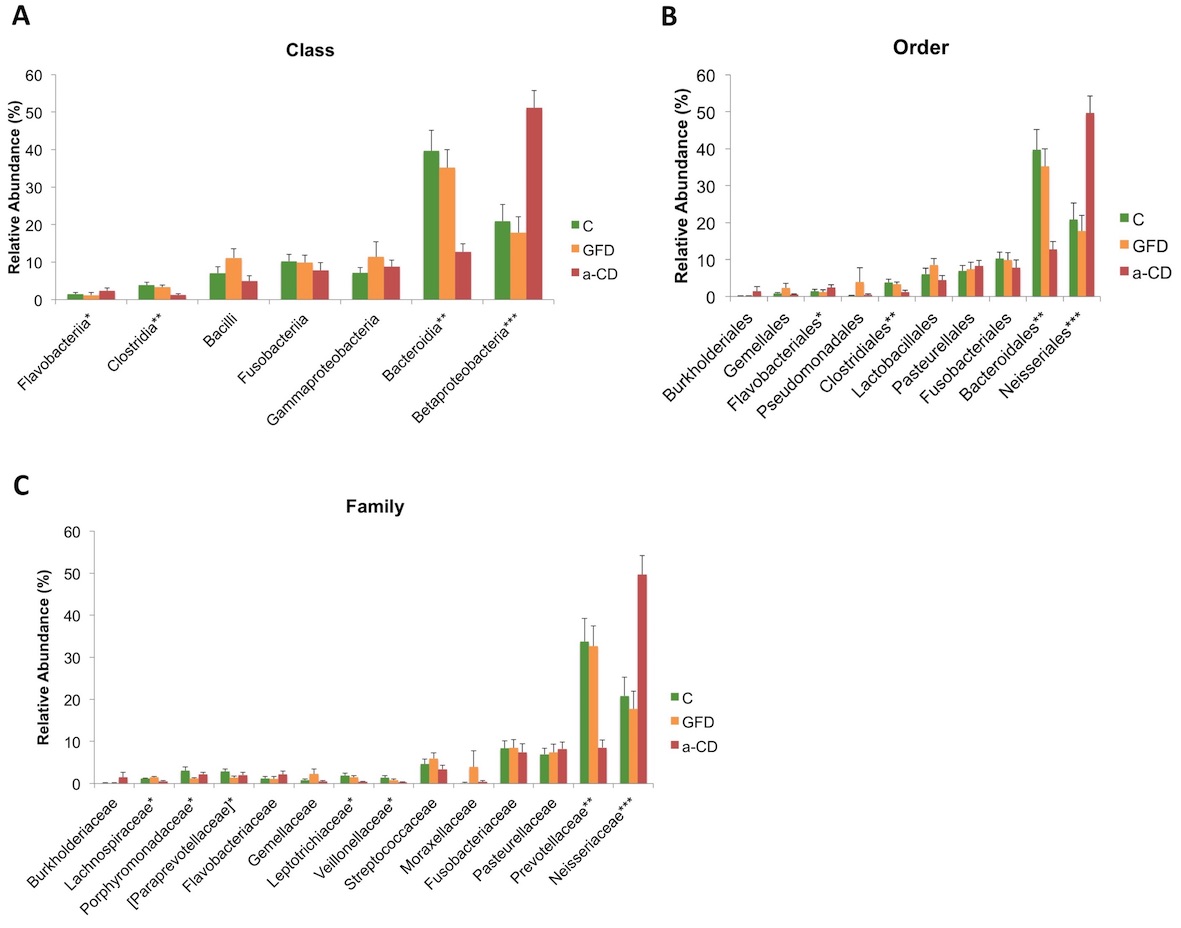


**Figure S2. Microbiome composition in the three study groups.** The barplots show the relative abundance (%) of taxonomic groups at class (A), order (B) and family (C) levels according to the Greengenes database v.13_8. Levels having abundance greater than 1% in at least one study group are reported. Error bars indicate standard error.


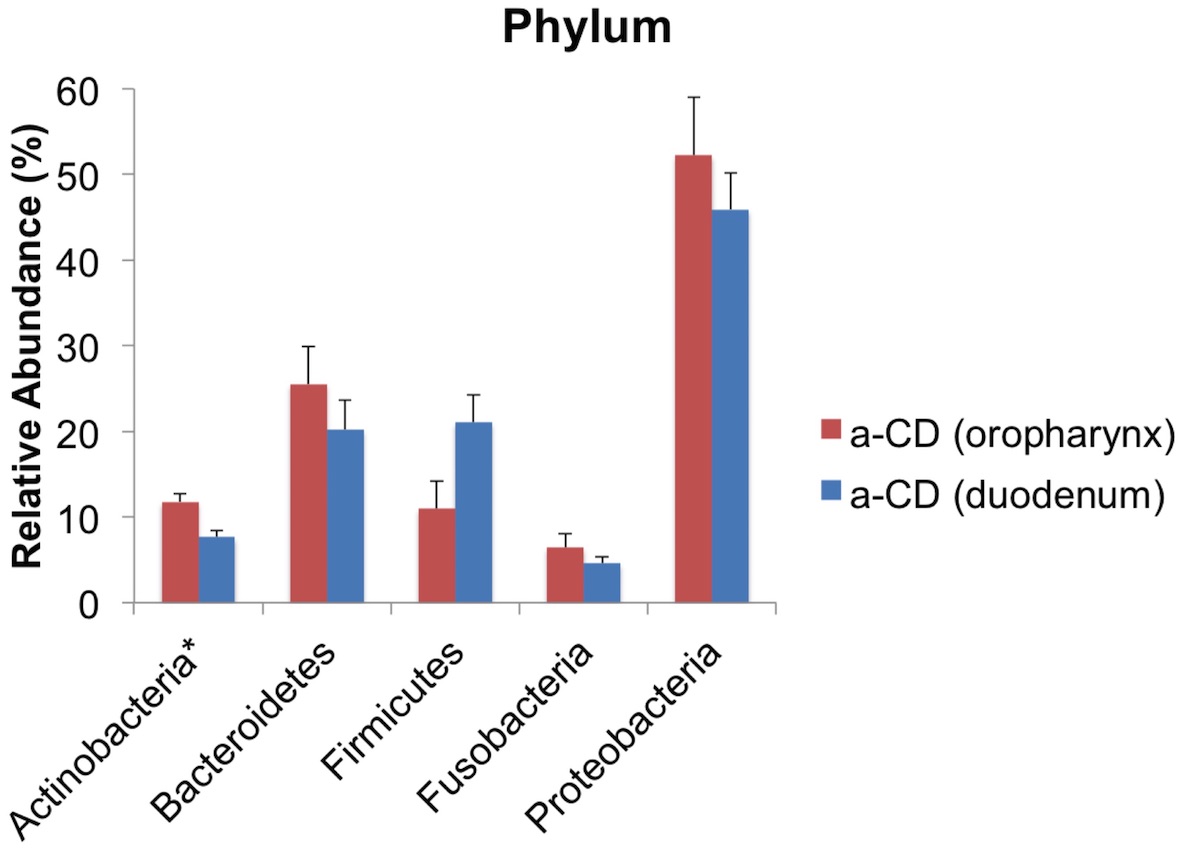


**Figure S3. Microbiome composition in the oropharynx and duodenum of a-CD patients.** The barplots show the relative abundance (%) of taxonomic groups at phylum level, according to the Greengenes database v.13_8. Phyla with an abundance exceeding 1% in at least one study group are reported. Error bars indicate standard error. Statistical significance between the two groups was assessed by Wilcoxon test. P-values were adjusted for multiple comparisons by FDR method. Asterisks refer to the significance of differences among the two groups (* adj.p<0.05).


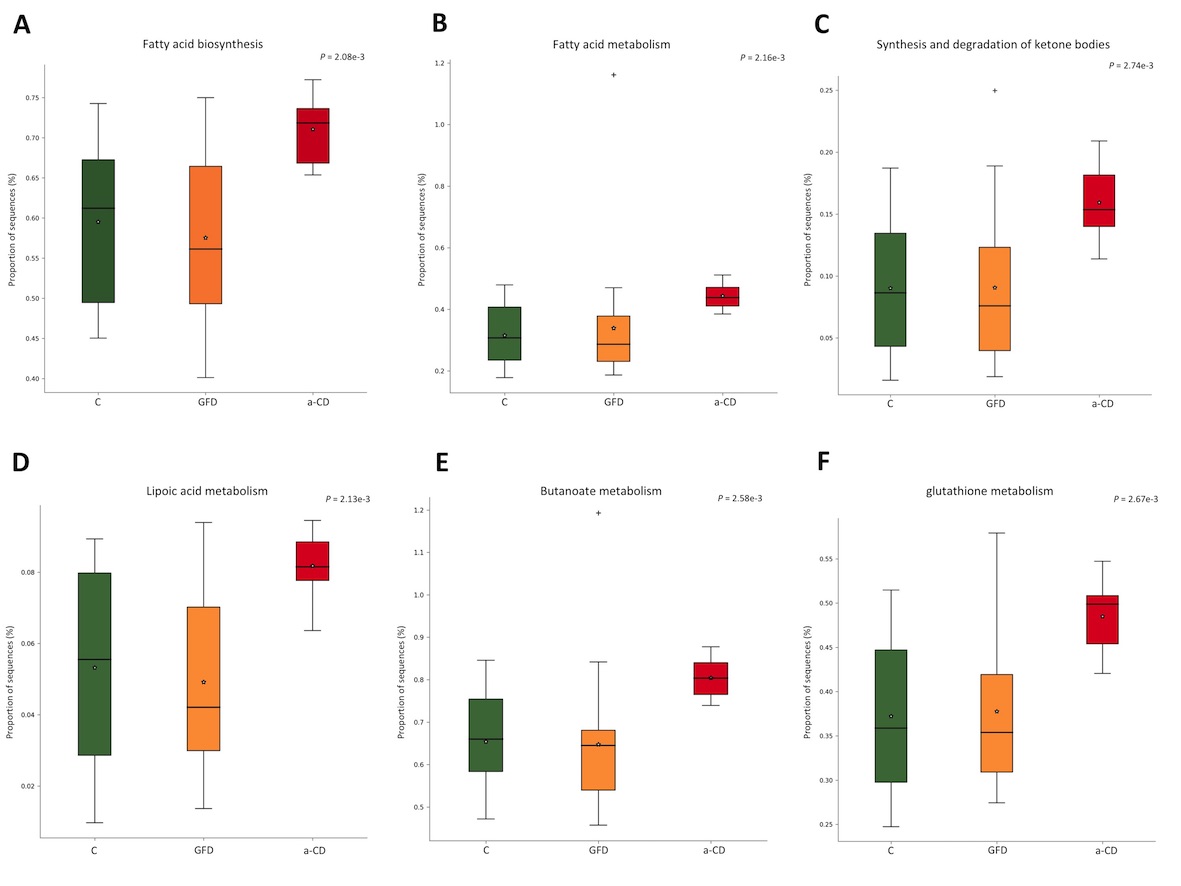


**Figure S4. Box plots showing the distribution in the proportion of specific KEGG pathways selected based on LDA score and literature assigned to the samples of the three groups.** Boxes indicate the IQR (75th to 25th of the data). The median value is shown as a line within the box and the mean value as a star. Whiskers extend to the most extreme value within 1.5*IQR. Outliers are shown as crosses. P-values calculated by Kruskal-Wallis test and multiple test corrected by Benjamini-Hochberg method are also shown. Boxplots were created by STAMP.


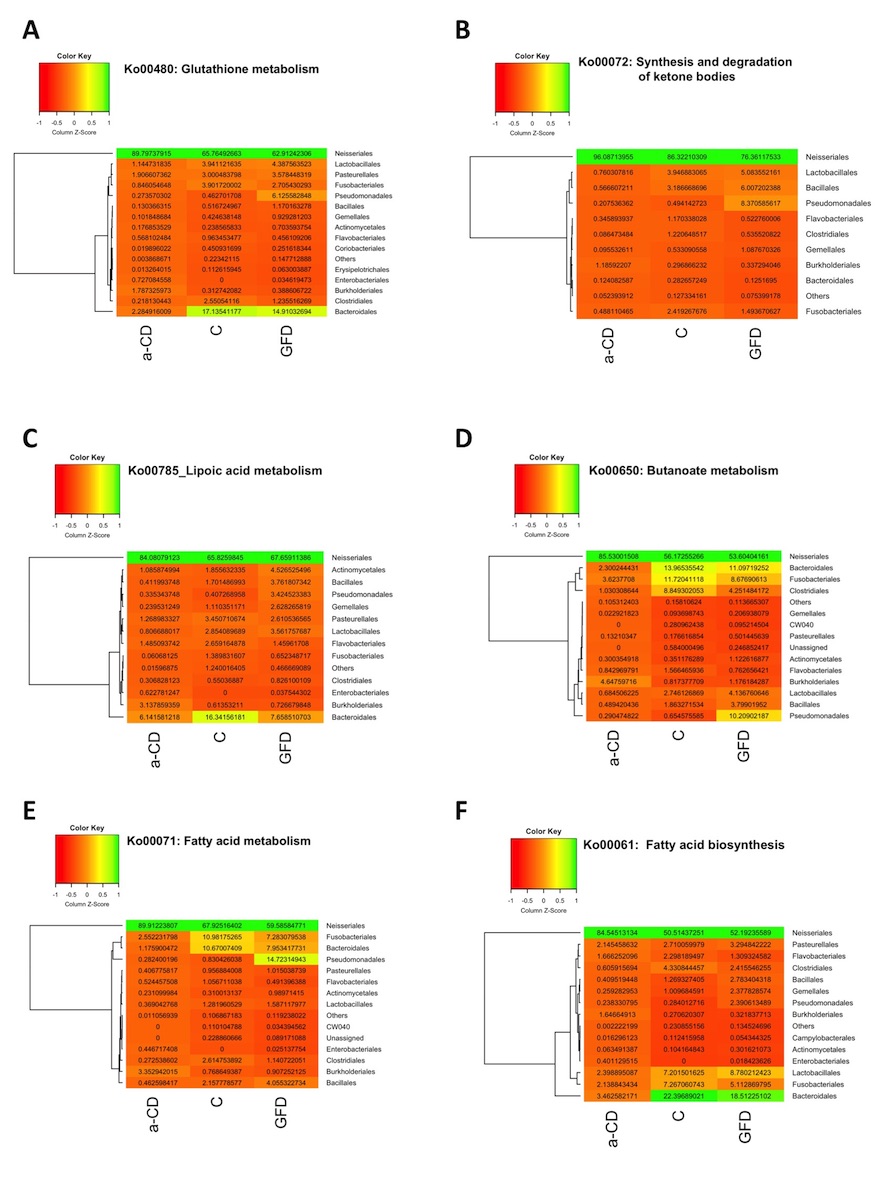


**Figure S5. Metagenome contribution of bacterial orders to gene functions. OTU contribution to some predicted functions (A-F).** Percentage values are reported within the heatmaps and the colors indicate the abundance and the different distribution of contributions among the three groups. “Others” indicates the order contributions present at <0.1% in all the three groups.
